# Is the version angle of the glenoid different in bone and cartilage? An MRI study

**DOI:** 10.3906/sag-1811-6

**Published:** 2019-10-24

**Authors:** Alper DEVECİ, Yahya Can DURA, Deniz SÖZMEN CILIZ, Guzelali ÖZDEMİR, Enver KILIÇ, Erman CEYHAN, Burak KULAKOĞLU, Sualp TURAN

**Affiliations:** 1 Department of Orthopedic Surgery, Ankara Numune Training and Research Center, University of Health Sciences, Ankara Turkey; 2 Department of Strategy, Ministry of Interior, Ankara Turkey; 3 Department of Radiology, Ankara Numune Training and Research Center, University of Health Sciences, Ankara Turkey

**Keywords:** Glenoid, glenoid version angle, shoulder

## Abstract

**Background/aim:**

To determine whether or not there is a difference between the version of the bone and cartilage surfaces of the glenoid. Axial magnetic resonance imaging (MRI) slices were examined in order to evaluate the measurements taken based on both the cartilage and bone joint surfaces.

**Materials and methods:**

A retrospective evaluation was made of the MRI scans of 182 patients. All of the reviewers independently measured the glenoid version angles of all of the patients from 1–182. The process was then repeated, with each reviewer taking second measurements of the angles from 1–182. Pearson correlation coefficient analysis was applied to evaluate the interaction and relationships between the measurements taken from the bone and cartilage. The intra- and interobserver interclass correlation coefficients (ICCs) were assessed. Analysis of variance was applied to determine any interobserver significant differences.

**Results:**

The mean glenoid version was determined as –3.58 ± 4.08° in the bone-based measurements and –5.81 ± 4.30° in the cartilage-based measurements. The cartilage- and bone-based measurements were found to have inter- and intraobserver reliability. A statistically significant difference was observed between the mean cartilage-based version and the mean bone-based version. Changes in the cartilage- and bone-based measurements were correlated; however, this change was not linear.

**Conclusion:**

The cartilage-based version showed a significant difference from the bone- based version. Therefore, in the preoperative planning and evaluation of glenoid-based pathologies, it would be more appropriate to evaluate both the bone and cartilage surface on MRI.

## 1. Introduction

The glenoid version is an important factor in the preoperative and intraoperative evaluation of glenohumeral instability and shoulder arthroplasty surgery. The current evaluation of the glenoid version is based on measurements made with computed axial tomography (CT) and 3-dimensional (3D) CT. However, there is no consensus with respect to the measurement methods and angular values [1–3].

By affecting the biomechanics of the glenohumeral joint, changes in the glenoid version may cause instability, arthropathy, and implant failure after arthroplasty procedures. Although the glenoid version is generally defined as retrovert, in some studies, antevert values, or a value close to 0, have been evaluated as normal [2–7]. This can be attributed to the measurements of the glenoid version being affected by several parameters [1–4,7–9]. Some of these are the twist effect within the surface of the glenoid joint, the coronal and sagittal position of the scapula, and the glenoid level at which the slice was taken. Despite different measurement techniques, measurement made using CT is the gold standard, based on the glenoid bone surface. In a magnetic resonance imaging (MRI) study of the patellofemoral joint [10], incompatibility between the deepest point of the cartilage trochlear groove and the deepest point of the bone trochlear groove was observed, and this was the source of inspiration for the current study. This raised the question of whether or not there are errors in the evaluation of the version of a joint surface covered in cartilage, when measurement is based on the bone.

In the absence of any pathological condition, despite the variation in such a wide range of version, the continuation of normal shoulder functions with no development of instability can be explained by a different approach. Does the cartilage surface change the bone surface version? The current study was planned to address this question. The aim of the study was to determine whether or not there was a difference between the version of the bone surface and the cartilage surface of the glenoid. Axial MRI slices were examined to be able to evaluate the measurements taken, based on both the cartilage and bone joint surfaces.

## 2. Materials and methods 

Approval for the study was granted by the Institutional Review Board of the Ankara Numune Training and Research Hospital (protocol number: E–18-1821). A retrospective evaluation was made of the MRI scans of 200 patients, aged 25–45 years, who presented at the polyclinic, between 2015 and 2017, with suspected shoulder pathology. Cases of partial and minor cuff tears, subacromial impingement, and superior labrum anterior posterior lesions type 1 and 2 were included in the study. Patients were excluded from the study if they had shoulder instability, osteoarthritis, rheumatoid arthritis, cuff tear arthropathy, cervical neuropathy, plexus pathology, previous shoulder surgery, bilateral shoulder complaints, or a gross effect of the relationship of the glenohumeral joint.

For measurements of the glenoid version, axial fat-suppressed proton density-weighted

(TR/TE: 2200/30 ms, matrix: 192 × 320, FOV: 18 × 18 cm, slice thickness: 4 mm) sequences were obtained. A total of 18 patients were excluded, as their cartilage and bone reference points could not be evaluated together on the same slice. Thus, evaluation was made of the MRIs of 182 patients, comprising 102 males and 80 females. The images were of 93 left shoulders and 89 right shoulders.

All of the MRIs were taken in our center, in the same position, with the same technique. The MRIs (Excite, GE Medical System, Wilwaukee, Wisconsin, USA) of the patients were taken with a 1.5-T unit using an extremity coil. The glenoid version of each patient was measured by 3 reviewers, independently of each other. These 3 reviewers were an orthopedic surgeon specialized in shoulder surgery, an orthopedic assistant physician, and a radiologist experienced with the musculoskeletal system. The measurements for each patient were performed twice by each reviewer.

Arthroscopy, which is one of the best evaluation methods for the shoulder joint and glenoid version, was not used here, which was one of the limitations of the study.

### 2.1. Measurements

The patients were numbered from 1–182. The file number of the patient to be measured for glenoid inclination and the level number of the MRI axial slice were defined by the first reviewer and the other 2 reviewers were informed about it. Hence, measurements were made using the same slice levels by all of the reviewers independently. Therefore, all of the reviewers independently measured the glenoid version angles of all of the patients from 1–182. The process was then repeated, with each reviewer taking second measurements of the angles from 1–182.

**Figure 1:**
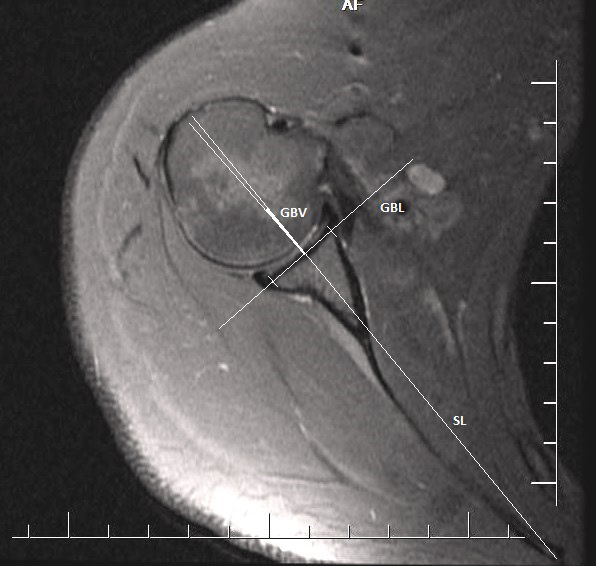
Bone-based glenoid version angle. GBL: glenoid bone line, SL: scapular line, GBV: glenoid bone version.

**Figure 2:**
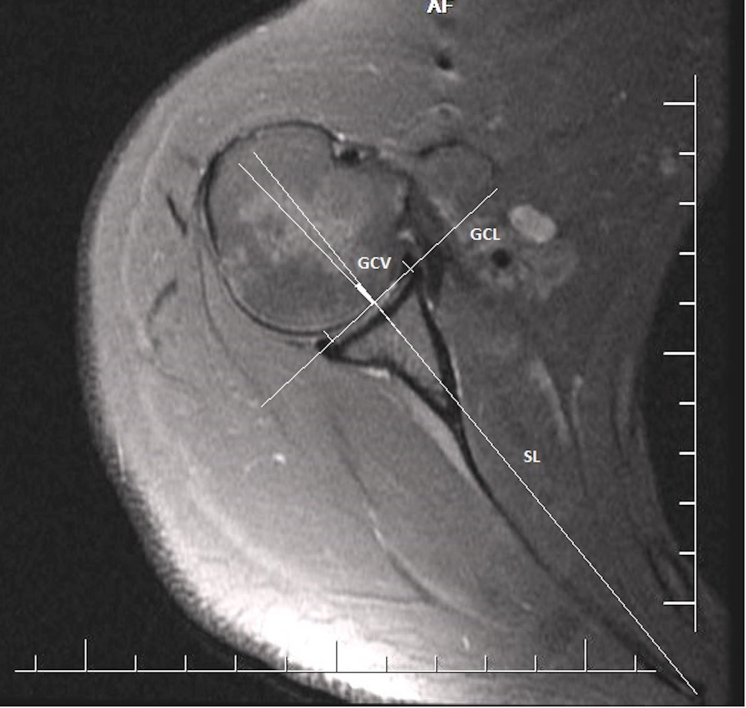
Cartilage-based glenoid version angle. GCL: glenoid cartilage line, SL: scapular line, GCV: glenoid cartilage version.

For all of the measurements, the MRI axial T2 sequences were evaluated. The slice from which the glenoid version measurement was to be made for each patient was determined by the senior reviewer using the Picture Archiving and Communication System. The glenoid version angle was measured using the Friedman method [11]. The first axial slices passing immediately inferior to the coracoid process were selected for the glenoid version measurement. For the bone-based glenoid version angle measurements, first the glenoid bone line (GBL) and scapular line (SL) were identified. The GBL was defined as the junction of the corner points of the anterior and posterior bone notches. The SL was formed from the line drawn joining the midpoint of the GBL and the most medial point of the scapula. The narrow angle between the SL and the GBL was evaluated as the glenoid bone version (GBV) angle (Figure 1). The same slice was used for the cartilage-based version measurement. The glenoid cartilage line (GCL) was formed by joining the corner points of the glenoid anterior and posterior cartilage. The SL was formed with a line drawn joining the midpoint of the GCL and the most medial point of the scapula. The narrow angle between the SL and the GCL was evaluated as the glenoid cartilage version (GCV) angle (Figure 2). Positive (+) values were evaluated as anteversion and negative (–) values as retroversion.

### 2.2. Statistical analysis

Statistical analyses of the data were performed using SPSS 22.0 software (IBM Corp., Armonk, NY, USA). All data were calculated as mean + standard deviation (SD). The intraobserver interclass correlation coefficients (ICCs) and interobserver ICCs were assessed. Conformity of the data to normal distribution was evaluated. By examining skewness-kurtosis values with significant values, according to the Kolmogorov–Smirnov test and the Shapiro–Wilk test, it was concluded that the series showed normal distribution. The analysis of variance (ANOVA) test was applied to determine any interobserver significant differences. To be able to determine the statistical relationship between the first and second measurements of bone and cartilage from each researcher, the dependent paired samples test was applied. The results were evaluated at a 95% confidence interval. P < 0.05 was accepted as statistically significant. The Pearson correlation coefficient analysis was applied to evaluate the interaction and relationships between the measurements taken from the bone and cartilage. 

## 3. Results

The mean age of the 182 patients in the study was 37 ± 6 years (range, 25–45 years). The mean, standard error, standard deviation, minimum, and maximum values related to the first and second measurements taken by each researcher were calculated. The mean glenoid version was determined as –3.58 ± 4.08° in the bone-based measurements and –5.81 ± 4.30° in the cartilage-based measurements.

The relationship between the first and second measurements from each researcher was evaluated using the paired samples test. When the first and second bone- and cartilage-based measurements were compared within themselves, no statistically significant difference was determined between the first and second measurements (P > 0.05; Table 1). When the bone- and cartilage-based measurements were compared with each other, a statistically significant difference was determined between the mean version values in both the first and second measurements (P < 0.05; Table 2). 

**Table 1:** Relationship between the first and second measurements of each observer.

Bone-based measurement	Mean	Standard deviation	Standard errorof the mean	P-value
Observer 1 first and second measurement	–3.90	0.01	0.01	0.130
Observer 2 first and second measurement	–3.68	0.03	0.03	0.260
Observer 3 first and second measurement	–3.17	0.03	0.03	0.057
Cartilage-based measurement				
Observer 1 first and second measurement	–5.93	0.11	0.11	0.105
Observer 2 first and second measurement	-–6.04	0.02	0.02	0.873
Observer 3 first and second measurement	–5.43	0.04	0.04	0.622

**Table 2:** Comparison of the observer bone and cartilage-based measurements.

Average bone-based measurement	ICC	P-value
Observer 1–2	0.947	0.000
Observer 1–3	0.835	0.000
Observer 2–3	0.873	0.000
Observer 1–3	0.920	0.000
Average cartilage-based measurement		
Observer 1–2	0.935	0.000
Observer 1–3	0.658	0.000
Observer 2–3	0.728	0.000
Observer 1–3	0.824	0.000

ICCs were used to determine intraobserver and interobserver variability. According to the results of the ICCs, there was a statistically significant concordance between each observer’s first and second measurements (Table 3), and their average bone- and cartilage-based measurements (Table 4).

**Table 3:** Intraobserver intraclass correlation coefficient.

Observer 1	ICC	P-value
Bone-based first and second measurement	0.999	0.000
Cartilage-based first and second measurement	0.958	0.000
Observer 2		
Bone-based first and second measurement	0.996	0.000
Cartilage-based first and second measurement	0.999	0.000
Observer 3		
Bone-based first and second measurement	0.997	0.000
Cartilage-based first and second measurement	0.996	0.000

**Table 4:** Interobserver intraclass correlation coefficient.

Average bone-based measurement	ICC	P-value
Observer 1–2	0.947	0.000
Observer 1–3	0.835	0.000
Observer 2–3	0.873	0.000
Observer 1–3	0.920	0.000
Average cartilage-based measurement		
Observer 1–2	0.935	0.000
Observer 1–3	0.658	0.000
Observer 2–3	0.728	0.000
Observer 1–3	0.824	0.000

ANOVA was applied to determine whether or not there was a difference between the interobserver measurements. The homogeneity test was applied first to the variances. The variances of the bone-based measurements were found to be homogenous and it was decided that variance analysis could be applied. As the cartilage-based measurements had a value of P < 0.05, variance analysis was not applied and one-way ANOVA was used (Table 5). No statistically significant difference was seen in the interobserver bone-based (P = 0.219) or cartilage-based (P = 0.393) measurements. A statistically significant difference in the 99% confidence interval was determined between the mean bone-based and cartilage-based measurements from all of the researchers (Table 6). 

**Table 5:** Evaluation of the interobserver measurement differences.

ANOVA					
		Sum of squares	Degrees of freedom	Mean square	P-value
Bone-basedmeasurement	Between groups	50.68	2	25.343	0.219
	Within groups	9049.381	544	16.666	
	Total	9100.067	546		
Cartilage-based measurement		1.870	2		0.393

**Table 6:** Evaluation of the differences between the mean bone-based measurements and mean cartilage-based measurements.

	Mean	Standard deviation	Standard errorof the mean	Degrees of freedom	P-value
Bone-based-Cartilage-based measurement	–2.207	3.632	0.155	546	0.000

The Pearson correlation coefficient analysis was applied to determine changes related to the bone-based and cartilage-based version measurements. A relationship between the mean bone and cartilage-based measurements was found at a moderate level of 0.626 with a significance of P < 0.05 (Table 7). 

**Table 7:** Correlation analysis to determine the relationship between the mean bone and cartilage-based measurements.

		Mean cartilage-based measurement	Mean of bone-based measurement
Mean cartilage-based measurement	Pearson correlation	1	0.626
	Sig. (2-tailed)		0.000
	N	546	546
Mean bone-based measurement	Pearson correlation	0.626	1
	Sig. (2-tailed)	0.000	
	N		546

**Correlation was significant at 0.01 (2-tailed).

To summarize the statistical results, the cartilage- and bone-based measurements were found to have inter- and intraobserver reliability. A statistically significant difference was observed between the mean cartilage-based and bone-based versions. The changes in the cartilage- and bone-based measurements were correlated. However, this change was not linear. When the effects on each other of the changes in the bone- and cartilage-based versions were examined, the effect on the bone-based version was low. Although the bone-based and cartilage-based version values were correlated, the relationship was not completely linear. In the increased bone-based retroversion values, the cartilage-bone retroversion difference was reduced.

## 4. Discussion

Correct evaluation of the glenoid version is important for many shoulder pathologies, and primarily arthritic glenohumeral joint and posterior instability [12–17]. Specifically, in anatomic total shoulder arthroplasty and reverse shoulder arthroplasty, glenoid preparation made by evaluation of the glenoid version is the most important stage [6,7,18–21]. In several studies related to glenoid version values, although there were differences in the lower and upper values, the glenoid joint surface was generally evaluated as retrovert [4,8,22]. In the current study, the mean values were –3.58 ± 4.08° in the bone-based measurements and –5.81 ± 4.30° in the cartilage-based measurements, and were thus, in retroversion. When considered in general, these results were consistent with the literature. Nevertheless, there were studies that reported very different limit values, associated with a series of factors, such as the difference in measurement techniques or the version difference between the glenoid upper and lower half [1–3,21,22].

The most important feature of the current study was that the glenoid version was evaluated with MRI based on the joint cartilage, which is the structure that determines the relationship and orientation of the joint. To the best of our knowledge, there have been no previous similar studies in the literature. The most significant finding of the study was that the glenoid bone- and cartilage-based version angles were statistically significantly different from each other. The glenoid cartilage-based measurements were observed to have higher retroversion angles than the bone-based measured values. Although the increase in the bone-based version angle was correlated with the increase in the cartilage-based version angle, this relationship was not linear. In particular, the increase in the cartilage-based version was lower when compared to the increase in the bone-based version. Consequently, the difference between the cartilage- and bone-based version values was observed to have decreased. This suggests that there could be a compensation mechanism for the stabilization of the glenohumeral joint. 

The source of inspiration for the current study was an MRI study of the anatomy of the patellofemoral joint published in 2002 [10]. In that study, it was revealed that the deepest points and the projections of the trochlear cartilage and bone grooves were not the same. Therefore, as the cartilage surface is the basic unit providing joint alignment and compatibility, when the deepest point of the trochlear groove was evaluated, it was determined on the basis of the cartilage, not the bone. From the starting point of this information, it was considered that rather than evaluating the bone version with CT, it would be more appropriate to evaluate the cartilage version with MRI. From the results of the current study, the cartilage-based version values were statistically significantly different from the bone-based version values (P < 0.05). Therefore, MRI is of value with respect to revealing the evident differences between the cartilage- and bone-based version angles. 

In the literature, the study of Anthony et al. on this subject is important [23]. They evaluated the difference between the bone and labral version angles using MRI. However, their work did not directly assess the joint surface. It is known that the labrum is a dynamic structure. It also has a large number of anatomical variations and is affected by many shoulder pathologies. For this reason, we think that it would be more accurate to base the cartilage instead of the labrum.

In the measurement of the glenoid version with CT, there are several different methods [1–3,8,11]. The Friedman method was used for measurements in the current study, as it is a practical and frequently used linear measurement method [11]. As radiological evaluation was made with MRI, this CT-based measurement method was more appropriate for the study. The method of taking axial slices in MRI, and CT and patient positions, are similar and the axial slices were evaluated in this study in the same way as the axial CT slices. The same reference points were used as in the CT measurements. Thus, it was aimed to eliminate differences between the CT and MRI measurements by using this linear measurement method. Although this may seem to be a deficient aspect of the current study, the bone-based version values determined on MRI were consistent with the CT-based version values in the literature [4,8,22]. Some limitations can be considered in the current study with respect to technique. One of these was the difficulty of the detailed evaluation of the cartilage on conventional MRI scans. A more powerful MRI device and technical support is required for the evaluation of cartilage, which is the primary component of joint compatibility. Nevertheless, although we used MRI with a 1.5 T resolution, we were able to evaluate the cartilage-bone distinction in 182 of 200 cases. Another limitation was the retrospective nature of the study. Nevertheless, this study has yielded 2 significant results. The first was that the bone- and cartilage-based versions measured on MRI were significantly different. The second was that although the cartilage-based version angles were higher than the bone-based version angles, when the retroversion values increased, the difference between the cartilage and bone measurements decreased. This second point can be attributed to the fact that in high glenoid bone-based version values, the increased version is balanced by a thickening of the cartilage tissue in the posterior. Therefore, in the measurement of the glenoid version, the cartilage reference points should be used rather than the bone reference points. 

In conclusion, the evaluation of the glenoid is important for several shoulder pathologies, especially in arthroplasty surgery. The glenoid version has a wide range in both bone- and cartilage-based measurements. It appears that cartilage-based retroversion is correlated with bone-based retroversion. However, the cartilage surface version showed a significant difference from the bone surface. Therefore, in the preoperative planning and evaluation of glenoid-based pathologies, it would be more appropriate to evaluate both the bone and cartilage surface on MRI.
